# Goal-directed therapy with continuous SvcO_2_ monitoring in pediatric cardiac surgery: the PediaSat single-center randomized trial

**DOI:** 10.1016/j.bjane.2025.844614

**Published:** 2025-03-28

**Authors:** Flavio M. Ferreira, David D. Araujo, Gustavo M. Dantas, Ligia Cristina C. Cunha, Suely P. Zeferino, Filomena B. Galas

**Affiliations:** Faculdade de Medicina da Universidade de São Paulo, São Paulo, SP, Brazil

**Keywords:** Hemodynamics, Lactate, Oxygen, Pediatrics, Postoperative care, Ventilator weaning

## Abstract

**Introduction:**

Low Cardiac Output Syndrome (LCOS) remains a significant perioperative challenge in pediatric cardiac surgery. This study evaluated whether a hemodynamic protocol aimed at optimizing continuous central venous Oxygen Saturation (SvcO_2_) using the PediaSat catheter could reduce postoperative complications in pediatric patients undergoing congenital heart surgery.

**Methods:**

Conducted at the Instituto do Coração in São Paulo, this randomized clinical trial compared a group receiving SvcO_2_-based goal-directed therapy via PediaSat (intervention) against conventional care (control). The main objective was assessing 24-hour lactate clearance post-surgery, with secondary outcomes including Vasoactive-Inotropic Score (VIS), Mechanical Ventilation (MV) duration, vasopressor use, and ICU/hospital stay lengths.

**Results:**

From July 13, 2014, to March 17, 2016, 391 patients were evaluated for eligibility. After applying inclusion and exclusion criteria, 65 patients were included and randomized ‒ 33 to the control group and 32 to the PediaSat group. There were no losses to follow-up in either group. Lactate clearance did not significantly differ between the intervention and control groups. However, the PediaSat group showed significantly shorter mechanical ventilation times, reduced vasopressor use, and shorter ICU stays. No significant differences were observed in hospital stay length or incidence of postoperative complications between the group.

**Conclusions:**

While optimizing SvcO_2_ did not affect overall lactate clearance, it was associated with shorter MV duration, decreased vasopressor need, and shorter ICU stays in pediatric cardiac surgery patients. These findings highlight the potential benefits of continuous SvcO_2_ monitoring in postoperative care.

## Introduction

Pediatric cardiac surgery has progressed remarkably in recent decades, allowing for increasingly complex procedures and improved survival rates for critically ill patients while reducing postoperative complications.^1^ Advances in surgical techniques, perioperative care, and understanding of congenital heart diseases have contributed to these improvements. However, one major perioperative challenge remains: Low Cardiac Output Syndrome (LCOS). It requires precise hemodynamic support to prevent and manage ensuing tissue hypoperfusion and organ dysfunction.^2^

Timely and accurate assessment of both cardiac output and tissue perfusion is critical in managing these patients, yet this remains difficult due to the unique physiological challenges of pediatric patients. Children have a limited capacity to increase stroke volume, relying more on heart rate to augment cardiac output. Additionally, their circulatory system is more sensitive to volume shifts and inotropic agents. The presence of congenital anomalies, such as intra- and extracardiac shunts, further complicates hemodynamic assessment.^3^

Multiple factors contribute to LCOS, including relative hypovolemia due to fluid shifts, myocardial dysfunction from ischemia-reperfusion injury, systemic vasodilation, and inflammatory responses triggered by cardiopulmonary bypass. The consequences of unchecked hemodynamic instability can be severe, leading to prolonged Intensive Care Unit (ICU) and hospital stays, increased healthcare costs, and adverse long-term outcomes, including higher morbidity and mortality rates.^4^^,^^5^

Goal-Directed Therapy (GDT) involves the use of predefined hemodynamic targets to guide therapeutic interventions, allowing for more tailored and precise management of fluid administration, inotropic support, and vasopressor use.^6^ By optimizing oxygen delivery and utilization, GDT aims to prevent tissue hypoxia and organ dysfunction.

One of the most promising approaches within GDT is continuous monitoring of central venous Oxygen Saturation (SvcO_2_). SvcO_2_ reflects the balance between oxygen delivery and consumption and serves as a surrogate for cardiac output and tissue perfusion.^7^ Continuous SvcO_2_ monitoring has been shown to improve outcomes in patients undergoing surgery for congenital heart disease by ensuring adequate oxygen delivery to tissues.^8^ In particular, GDT guided by SvcO_2_ has demonstrated improved survival rates and reduced organ dysfunction in both septic patients and those undergoing complex cardiac procedures.^9^

Studies have shown that failure to optimize SvcO_2_ postoperatively is associated with higher rates of organ dysfunction and mortality. Specifically, in pediatric cardiac surgery, SvcO_2_ levels below 68% and lactate levels above 3 mmoL.L^-1^ during cardiopulmonary bypass have been linked to increased morbidity and mortality.^4^

This study tests the hypothesis that GDT aimed at optimizing SvcO_2_ through continuous monitoring using the PediaSat catheter can provide superior tissue perfusion and reduce postoperative complications when compared to conventional hemodynamic management in pediatric patients undergoing congenital cardiac surgery. By providing real-time data, continuous SvcO_2_ monitoring may allow for earlier detection of hemodynamic instability and more timely interventions, potentially improving patient outcomes.^10^^,^^11^

## Methods

### Study design

This prospective, randomized, controlled trial assessed the efficacy of goal-directed hemodynamic optimization in pediatric patients undergoing cardiac surgery with cardiopulmonary bypass. The protocol was approved by the Ethics Committee for Research Project Analysis (CAPPesq) at the HC-FMUSP, University of São Paulo, and registered at ClinicalTrials.gov (identifier NCT03469440).

### Study population

The study included pediatric patients under 14 years of age diagnosed with congenital cardiac anomalies that required corrective or palliative surgery involving cardiopulmonary bypass. All participants were classified according to the Risk Adjustment for Congenital Heart Surgery (RACHS-1) system. Patients were excluded if they presented anatomical limitations preventing central venous catheter placement, significant arrhythmias, hemodynamic instability requiring norepinephrine doses higher than 0.5 µg.kg^-1^.min^-1^, active malignancies, or if they were candidates for urgent surgery or transplantation.

Informed consent was obtained from the legal guardians or parents of all participants, following institutional and national ethical guidelines. Assent from the children was not obtained due to their young age, in accordance with ethical guidelines.

### Patient enrollment and randomization

**Figure 1:**
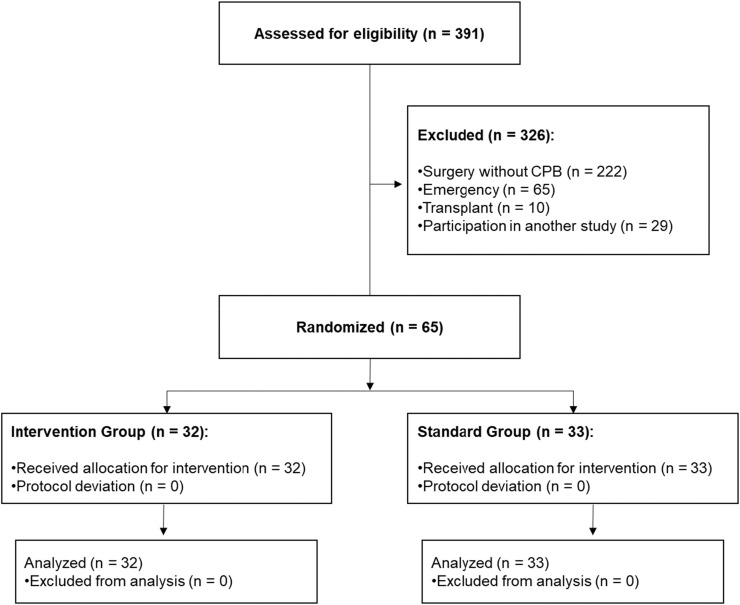
CONSORT Flow diagram.

Between July 13, 2014, and March 17, 2016, 391 patients were evaluated for eligibility. After assessing the inclusion and exclusion criteria, 65 patients were included in the study and randomized ‒ 33 patients to the control group and 32 to the PediaSat group (Fig. 1). There were no losses to follow-up in either group.

### Randomization

Participants were randomly assigned to the control or PediaSat group using a computer-generated sequence with variable block sizes. Allocation concealment was ensured using sequentially numbered, opaque, sealed envelopes prepared by an independent researcher not involved in patient recruitment. Randomization was performed after patient eligibility was confirmed and consent was obtained. Blinding was not feasible due to the nature of the intervention, which is acknowledged as a study limitation.

### Instrumentation and monitoring technology

The primary intravenous fluids used were balanced crystalloid solutions. Colloids, such as Voluven and albumin, were administered selectively based on clinical indications. The volume of fluids administered was recorded for both groups, with the PediaSat group receiving a significantly higher volume, as detailed in the results. Vasopressors, including norepinephrine, dopamine, and epinephrine, were utilized at the discretion of the attending anesthesiologist.

Patients in the intervention group were managed using the PediaSat oximetry catheter, which provided continuous central venous Oxygen Saturation (SvcO₂) monitoring. The catheter was inserted through the right internal jugular or subclavian vein and advanced to the superior vena cava or right atrium, with proper placement confirmed by postoperative chest radiography. Placement depth was determined based on patient size and imaging to ensure accuracy. This catheter employs fiber-optic technology, allowing real-time monitoring to guide fluid administration and inotropic support.

Despite its advantages, the PediaSat catheter posed some challenges, particularly in small children, due to technical difficulties during placement. Potential complications included catheter malposition, risks of infection, and mechanical issues. Proper positioning and interpretation of the data required trained personnel, emphasizing the importance of expertise for optimal utilization.

### Intervention and control groups

In the control group, standard care included monitoring of intermittent clinical parameters such as heart rate, blood pressure, Central Venous Pressure (CVP), capillary refill time, skin color, extremity temperature, and lactate levels at predefined intervals. Hemodynamic management was guided by clinical judgment and physical examination findings. In the intervention group, Goal-Directed Therapy (GDT) was applied using continuous SvcO_2_ monitoring via the PediaSat catheter. Therapeutic interventions were adjusted in real time to maintain SvcO_2_ above 65% in acyanotic patients and above 55% in cyanotic patients, ensuring tailored management of fluid and inotropic therapy.

### Outcomes

The primary outcome was 24-hour postoperative lactate clearance, evaluated as both a continuous variable, representing the percentage reduction in lactate levels within the first 24 hours, and a categorical variable, defined as the proportion of patients achieving a clearance rate above 30%. Secondary outcomes included the incidence of complications such as renal failure (as defined by pRIFLE criteria), vasoplegia, low cardiac output syndrome, arrhythmias, infections, and significant postoperative bleeding. Additional secondary outcomes included the duration of mechanical ventilation, inotropic support, vasopressor use (measured using the Vasoactive-Inotropic Score, VIS), and ICU and hospital stays.

### Measurements

Hemodynamic parameters, including SvcO_2_, arterial and venous lactate levels, Mean Arterial Pressure (MAP), Heart Rate (HR), and urine output, were continuously monitored or recorded at predefined intervals. SvcO_2_ was continuously recorded hourly in the intervention group. Lactate levels were measured at induction, post-surgery, and at intervals of 6, 12, 18, and 24 hours postoperatively. Other hemodynamic variables, such as HR, MAP, and CVP, were recorded hourly, while urine output was continuously monitored and recorded hourly.

### Statistical analysis

The sample size of 65 patients (33 in the control group and 32 in the intervention group) was determined based on prior studies that investigated continuous central venous oxygen saturation monitoring in pediatric cardiac surgeries. Crowley et al. demonstrated that prolonged SvcO_2_ desaturation was associated with adverse outcomes, while Ranucci et al. validated the PediaSat catheter for continuous monitoring in pediatric patients. These findings supported the appropriateness of the chosen sample size for comparing both primary and secondary outcomes in this study.

Data were analyzed on an intention-to-treat basis using SPSS version 18.0 (SPSS Inc., Chicago, IL, USA). Continuous variables were summarized as means with standard deviations for normally distributed data or medians with interquartile ranges for non-normally distributed data. Categorical variables were presented as frequencies and percentages. The rationale for statistical tests was based on the characteristics of the data. The Student's *t*-test was used to compare means between groups for continuous variables with a normal distribution, as confirmed by visual inspection of histograms and the Shapiro-Wilk test. For continuous data that were not normally distributed, the Mann-Whitney *U* test was applied, offering a robust non-parametric alternative. Comparisons of categorical variables between groups were conducted using the Chi-Square test, with Fisher's exact test employed for analyses involving small sample sizes to ensure robustness.

Confidence Intervals at 95% were calculated for all comparisons to provide an estimate of the precision of observed differences, enhancing the interpretability of the findings. Effect sizes were also calculated to complement p-values, offering insight into the magnitude of differences between groups. A p-value of less than 0.05 was considered statistically significant. The inclusion of confidence intervals alongside p-values was intended to ensure that the interpretation of findings extended beyond statistical significance, emphasizing clinical relevance.

## Results

### Baseline characteristics

The baseline demographic and clinical characteristics of the patients are presented in Table 1. Both groups were similar in terms of age, weight, gender distribution, and type of congenital heart disease.

**Table 1:** Baseline and demographic characteristics of patients.

Variable	Control (n = 33)	PediaSat (n = 32)	p-value
**Age (years), mean ± SD**	1.33 ± 2.41	2.44 ± 3.03	0.108^d^
**Gender (Male)**	18 (54.5%)	20 (62.5%)	0.515^a^
**Race**			1.000^b^
White	31 (93.9%)	30 (93.8%)	
Black	2 (6.1%)	2 (6.3%)	
**BMI (kg.m^-2^), median (IQR)**	15 (13 ‒ 16)	15 (13 ‒ 17)	0.689^e^
**RACHS-1**			0.515^c^
Score 1	1 (3%)	1 (3.1%)	
Score 2	16 (48.5%)	20 (62.5%)	
Score 3	14 (42.4%)	8 (25%)	
Score 4	2 (6.1%)	3 (9.4%)	
**LVEF (%)**	67 ± 10.59	72.29 ± 9.69	0.069^d^
**RV Dysfunction**	3 (9.1%)	3 (9.4%)	1.000^b^
**Pulmonary Hypertension**	9 (27.3%)	5 (15.6%)	0.253^a^
**Congenital Heart Disease Types, n (%)**			0.320^a^
Left-to-Right Shunt	16 (49%)	13 (41%)	
Right-to-Left Shunt	12 (36%)	9 (28%)	
Single Ventricle	1 (3%)	0 (0%)	
Obstructive Lesions	3 (9%)	8 (25%)	
Conotruncal	1 (3%)	1 (3%)	
Other	0 (0%)	1 (3%)	
**Previous Cardiac Surgery**	9 (27.3%)	13 (40.6%)	0.255^a^
**PRISM III (ICU admission), mean ± SD**	9.13 ± 4.11	7.72 ± 3.34	0.154^d^

Source: Ferreira, 2018.^14^

a Chi-Square test;

b Fisher's exact test;

c Likelihood ratio test;

d Student's *t*-test;

e Mann-Whitney test. SD, Standard Deviation; BMI, Body Mass Index; IQR, Interquartile Range; RACHS-1, Risk Adjustment in Congenital Heart Surgery; LVEF, Left Ventricular Ejection Fraction; RV, Right Ventricle; PH, Pulmonary Hypertension.

### Primary outcome: lactate clearance

Lactate clearance rates did not differ significantly between the PediaSat and control groups. The primary outcome was analyzed as both a continuous variable (percentage reduction in lactate levels) and a categorical variable (proportion of patients with clearance > 30%). At 24 hours postoperatively, the proportion of patients achieving lactate clearance above 30% was 59.4% in the PediaSat group compared to 60.6% in the control group (p = 0.919). Lactate levels were measured at baseline, intraoperatively, and at 6-, 12-, and 24-hour intervals postoperatively (Table 2).

**Table 2:** Lactate levels during intraoperative and Intensive Care Unit.

Time Point	Control Group (n = 33)	PediaSat Group (n = 32)	p-value	95% CI (Control Group)	95% CI (PediaSat Group)
Onset of ECC (mmoL.L^-1^)	1.85 ± 1.49	1.99 ± 1.58	0.686	[1.34, 2.36]	[1.44, 2.54]
End of ECC (mmoL.L^-1^)	4.69 ± 1.94	3.61 ± 1.47	0.045^a^	[4.03, 5.35]	[3.10, 4.12]
ICU Admission (mmoL.L^-1^)	3.93 ± 2.64	2.56 ± 1.51	0.048^a^	[3.03, 4.83]	[2.04, 3.08]
6 hours (mmoL.L^-1^)	3.63 ± 1.68	3.29 ± 2.58	0.570	[3.06, 4.20]	[2.40, 4.18]
12 hours (mmoL.L^-1^)	2.09 ± 1.06^b^	2.43 ± 2.02	0.460	[1.69, 2.57]	[1.73, 3.13]
18 hours (mmoL.L^-1^)	2.13 ± 1.29^b^	2.17 ± 1.67	0.900	[1.69, 2.57]	[1.59, 2.75]
24 hours (mmoL.L^-1^)	2.87 ± 1.35^a^^,^^b^	1.27 ± 0.85^a^	0.030^a^	[2.41, 3.33]	[0.98, 1.57]
Clearance > 30% (6 hours)	15.2% (5/33)	12.5% (4/32)	1.000**	‒	‒
Clearance > 30% (12 hours)	45.5% (15/33)	46.9% (15/32)	0.909	‒	‒
Clearance > 30% (18 hours)	54.5% (18/33)	50% (16/32)	0.714	‒	‒
Clearance > 30% (24 hours)	60.6% (20/33)	59.4% (19/32)	0.919	‒	‒

Repeated measures analysis of variance.

a p < 0.05 compared to the CONTROL group.

b p < 0.05 compared to the ADM moment. ECC, Extracorporeal Circulation; SD, Standard Deviation.

### Secondary outcomes

The PediaSat group had shorter mechanical ventilation, less vasopressor use, and a shorter ICU stay compared to the control group. Although lower, the mean VIS score in the PediaSat group was not statistically significant (Table 3).

**Table 3:** Secondary outcomes between groups.

Outcome	Control group (n = 33)	Pediasat group (n = 32)	p-value	95% CI (control)	95% CI (PediaSat)
Mechanical ventilation duration (hours)	64 ± 19.33	22 ± 85.10	0.005^c^	[21.46 – 27.52]	[72.55 – 98.21]
Vasopressor use duration (hours)	85.27 ± 47.72	19.28 ± 113.83	0.021^c^	[11.79 – 26.76]	[68.11 – 102.43]
VIS score (mean±sd)	60.61 ± 79.46	45.14 ± 29.47	0.266^d^	[27.17 – 94.05]	[32.22 – 58.06]
ICU stay (days)	8 ± 13.64	6 ± 9.84	0.030^d^	[8.87 – 12.98]	[10.82 – 13.79]
Hospital stay (days)	24 ± 21.61	23 ± 13.73	0.503^d^	[27.16 – 33.68]	[23.16 – 27.30]
Renal failure, n (%)	6 (18.2%)	3 (9.4%)	0.479^a^		
Vasoplegia, n (%)	11 (34.4%)	5 (15.6%)	0.083^a^		
LCOS, n (%)	6 (18.2%)	3 (9.4%)	0.475^a^		
Arrhythmias, n (%)	7 (21.2%)	5 (15.6%)	0.562^a^		
Infections, n (%)	10 (30.3%)	11 (34.4%)	0.726^a^		
Bleeding, n (%)	3 (9.1%)	0 (0%)	0.238^a^		
Reoperation, n (%)	6 (18.2%)	1 (3.1%)	0.105^a^		

Source: Ferreira, 2018.^14^

a Chi-Square test. ^b^ Fisher's exact test.

c Likelihood ratio test.

d Student's t-test. ^e^ Mann-Whitney test.

The intervention group received more intravenous fluids than the control group (24 mL.kg^-1^^13^, ^14^, ^15^, ^16^, ^17^, ^18^, ^19^, ^20^, ^21^, ^22^, ^23^, ^24^, ^25^, ^26^, ^27^, ^28^, ^29^ vs. 17 mL.kg^-1^^9^, ^10^, ^11^, ^12^, ^13^, ^14^, ^15^, ^16^, ^17^, ^18^, ^19^, ^20^, ^21^, p < 0.001). Both groups primarily used balanced crystalloid solutions, with occasional use of colloids. Vasopressor administration, including norepinephrine, dopamine, and epinephrine, was similar between groups, reflecting consistent application of individualized clinical judgment.

### Hemodynamic and perfusion variables

PediaSat group had slightly higher heart rates and mean arterial pressures postoperatively, no significant differences in central venous pressure were noted between the two groups.

### Postoperative complications

The incidence of postoperative complications such as renal failure, vasoplegia, arrhythmias, bleeding, infections, and reoperations was similar between the groups (Table 3).

### Distribution of congenital heart diseases

The distribution of congenital heart diseases among the two groups is illustrated in Table 4.

**Table 4:** Distribution of congenital heart diseases among groups.

Congenital Heart Disease	Standard (n)	Intervention (n)	Standard (%)	Intervention (%)	p-value
Left-to-right shunt	16	14	49%	42%	0.805^a^
Right-to-left shunt	11	8	33%	24%	0.587^a^
Single ventricle	1	0	3%	0%	1.000^a^
Obstructive lesions	3	8	9%	24%	0.186^a^
Conotruncal	1	1	3%	3%	1.000^a^
Other	1	2	3%	6%	0.558^a^

Source: Ferreira, 2018.^14^

a The p-values were calculated using the Chi-Square test for each category of congenital heart disease.

## Discussion

### Summary of findings

This randomized controlled trial evaluated the impact of goal-directed hemodynamic management using continuous SvcO_2_ monitoring via the PediaSat catheter in pediatric cardiac surgery. While 24-hour lactate clearance did not differ significantly between the intervention and control groups, notable reductions in mechanical ventilation duration, vasopressor use, and ICU length of stay were observed in the PediaSat group. These results indicate that continuous SvcO_2_ monitoring enables precise interventions, improving postoperative recovery. Although mean VIS scores were lower in the PediaSat group, this difference was not statistically significant.

Despite the data being collected between 2014 and 2016, the principles of hemodynamic optimization and SvcO_2_ monitoring remain highly relevant. Despite technological advances, continuous SvcO_2_ monitoring is still not standard practice in many centers, underscoring the relevance of these findings. This study highlights a persistent gap in perioperative pediatric cardiac care, underscoring the need for broader clinical adoption and further research.

### Interpretation of results

The lack of significant differences in lactate clearance between groups can be attributed to several factors. Lactate clearance is influenced by multiple variables, including tissue perfusion, oxygen delivery, metabolic rates, and hepatic clearance.^15^^,^^16^ While continuous SvcO_2_ monitoring likely optimized oxygen delivery and reduced anaerobic metabolism, individual variability in hepatic function, inflammatory responses, and baseline adequacy of lactate levels may have limited the ability to detect differences. This underscores the limitations of using lactate clearance as a sole indicator of clinical improvement.

Despite this, lower absolute lactate levels at specific postoperative time points in the PediaSat group suggest improved tissue perfusion and oxygen utilization. The observed reductions in mechanical ventilation duration and ICU stay further indicate that continuous SvcO_2_ monitoring enabled earlier detection and correction of hemodynamic instability. Maintaining optimal SvcO_2_ levels likely minimized tissue hypoxia, aiding faster recovery.

Reduced vasopressor use and shorter ventilation highlight continuous SvcO_2_ monitoring's role in improving hemodynamic stability. Although lower VIS scores in the PediaSat group were not statistically significant, they align with reduced dependence on inotropic and vasopressor support, reinforcing the potential clinical benefits of this approach.

### Comparison with previous studies

Our findings align with prior research demonstrating the benefits of Goal-Directed Therapy (GDT) and continuous SvcO_2_ monitoring in pediatric cardiac surgery. Rossi et al.^3^ reported that goal-directed medical therapy improved outcomes after congenital heart surgery by tailoring hemodynamic management. Similarly, Ranucci et al.^4^ showed that low SvcO_2_ and elevated lactate levels during cardiopulmonary bypass were associated with worse outcomes, highlighting the prognostic value of these parameters.

Studies have shown that optimized hemodynamic management can reduce the need for mechanical ventilation and vasopressor support.^17^^,^^18^ The trend toward lower VIS scores observed in this study is consistent with previous research, suggesting that continuous SvcO_2_ monitoring reduces the need for vasoactive medications. These findings also suggest potential reductions in morbidity and healthcare costs associated with shorter ICU stays and less invasive interventions.

### Clinical implications

Implementing continuous SvcO_2_ monitoring within a GDT framework offers several clinical advantages.^19^^,^^20^ Real-time data enable earlier detection of hemodynamic deterioration, allowing timely interventions such as fluid boluses, inotropic adjustments, or transfusions. This approach minimizes reliance on delayed or nonspecific intermittent measurements.^21^

Reducing mechanical ventilation duration is particularly significant, as prolonged ventilation is associated with increased risks of ventilator-associated pneumonia and lung injury. Shorter ventilation times enhance recovery and reduce complications.^19^^,^^20^ Similarly, reducing ICU stay lowers resource utilization and decreases risks of nosocomial infections, highlighting the broader benefits of continuous SvcO_2_ monitoring in optimizing postoperative care.^21^

### Challenges and limitations of SvcO_2_ monitoring

While promising, continuous SvcO_2_ monitoring presents challenges. Accurate measurements depend on proper catheter placement, which can be technically challenging in small children or patients with complex anatomy.^5^ Potential measurement errors from malposition, calibration issues, or ambient light interference also require mitigation through strict protocols.^6^ In this study, pre-insertion calibration, radiographic placement verification, and staff training minimized inaccuracies, ensuring consistency in SvcO_2_ data interpretation.

Despite these measures, SvcO_2_ monitoring alone does not provide a comprehensive assessment of hemodynamic status. Factors such as anemia, arterial oxygenation, and oxygen consumption variability affect SvcO_2_ independently of cardiac output.^18^ To address this, SvcO_2_ monitoring should complement other clinical assessments and hemodynamic parameters.

### Practicality and feasibility of implementation

Scaling continuous SvcO_2_ monitoring in broader settings requires consideration of cost-effectiveness, particularly in resource-limited environments. The PediaSat catheter and associated equipment involve substantial financial investment. However, reductions in ICU stay, mechanical ventilation duration, and vasopressor use could offset these costs. Strategies such as bulk procurement, reusable equipment, and training programs for healthcare professionals could enhance feasibility.

Further studies evaluating the cost-effectiveness and scalability of continuous SvcO_2_ monitoring across diverse clinical settings are essential to guide broader adoption. By addressing these challenges, continuous SvcO_2_ monitoring could become a standard of care in pediatric cardiac surgery.

### Limitations of the study

This study has limitations that should be considered. Although the sample size is consistent with similar studies, it may have limited statistical power to detect subtle differences in outcomes like VIS scores. Additionally, as a single-center study conducted in a specialized tertiary facility, the findings may not be generalizable to diverse clinical settings with varying resources and expertise.

Moreover, this study provides a focused evaluation of continuous SvcO_2_ monitoring but lacks the broader applicability of multicenter trials. Larger studies, such as those by Ranucci et al. and Tweddell et al., highlight the need for sufficient sample sizes and multicenter collaboration to confirm findings.^4^^,^^7^ Sankar et al.'s trial in pediatric septic shock emphasizes variability across diverse settings.^9^ Future research should validate these findings in larger, multicenter cohorts to enhance generalizability and clinical evidence.

The lack of blinding may have introduced bias, potentially influencing care delivery. However, standardized protocols and blinded data analysis were employed to minimize these effects. Finally, this study did not assess long-term outcomes, such as neurodevelopmental status or quality of life, emphasizing the need for extended follow-up in future research.

### Future directions

Future research should validate these findings in larger, multicenter trials to enhance generalizability and address the variability in outcomes observed across diverse settings. Incorporating advanced monitoring techniques, such as Near-Infrared Spectroscopy (NIRS), could offer a more comprehensive evaluation of tissue perfusion.^25^ Studies could also explore the cost-effectiveness of implementing continuous SvcO_2_ monitoring, considering the potential reductions in ICU stay and mechanical ventilation duration.^26^^,^^27^ Personalized hemodynamic targets based on patient-specific risk profiles could further optimize goal-directed therapy, advancing its clinical utility.^28^, ^29^, ^30^

## Conclusion

In pediatric cardiac surgery patients, a hemodynamic management strategy guided by continuous monitoring of central venous Oxygen Saturation (SvcO_2_) was associated with significant clinical benefits, including shorter mechanical ventilation duration, reduced vasopressor requirements, and decreased ICU length of stay. Although no significant differences were observed in 24-hour lactate clearance between the intervention and control groups, the observed improvements in secondary outcomes highlight the potential clinical utility of this technology in optimizing tissue oxygen delivery. These findings suggest that the integration of real-time SvcO_2_ monitoring into perioperative care protocols may enhance postoperative recovery by enabling earlier detection and intervention in cases of hemodynamic instability.

## Authors’ contributions

Flavio M. Ferreira: Study conception and design, data analysis.

David D. Araujo: Data collection, manuscript drafting.

Gustavo M. Dantas: Data collection, critical manuscript review.

Ligia Cristina C. Cunha: Study supervision, results interpretation.

Suely P. Zeferino: Literature review, research protocol development.

Filomena B. Galas: Overall project coordination, final manuscript approval.

## Conflicts of interest

The authors declare no conflicts of interest.

## References

[1] Martin G., Jonas R. (2018). Surgery for Congenital Heart Disease: Improvements in Outcomes. Am J Perinatol.

[2] Epting C.L., McBride M.E., Wald E.L., Costello JM. (2015). Pathophysiology of Post-Operative Low Cardiac Output Syndrome. Curr Vasc Pharmacol.

[3] Rossi A.F., Khan D.M., Hannan R., Bolivar J., Zaidenweber M., Burke R. (2005). Goal-directed medical therapy and point-of-care testing improve outcomes after congenital heart surgery. Intensive Care Med.

[4] Ranucci M., Isgrò G., Carlucci C. (2010). Central venous oxygen saturation and blood lactate levels during cardiopulmonary bypass are associated with outcome after pediatric cardiac surgery. Crit Care.

[5] Pagowska-Klimek I., Pychynska-Pokorska M., Krajewski W., Moll JJ. (2011). Predictors of long intensive care unit stay following cardiac surgery in children. Eur J Cardiothorac Surg.

[6] Ramsingh D., Hu H., Yan M. (2021). Perioperative Individualized Goal Directed Therapy for Cardiac Surgery: A Historical-Prospective, Comparative Effectiveness Study. J Clin Med.

[7] Tweddell J.S., Ghanayem N.S., Mussatto K.A. (2007). Mixed Venous Oxygen Saturation Monitoring After Stage 1 Palliation for Hypoplastic Left Heart Syndrome. Ann Thorac Surg.

[8] Law M.A., Benscoter A.L., Borasino S. (2022). Inferior and Superior Vena Cava Saturation Monitoring After Neonatal Cardiac Surgery*. Pediatr Crit Care Med.

[9] Sankar J., Sankar M.J., Suresh C.P., Dubey N.K., Singh A. (2014). Early Goal-Directed Therapy in Pediatric Septic Shock: Comparison of Outcomes “With” and “Without” Intermittent Superior Venacaval Oxygen Saturation Monitoring. Pediatr Crit Care Med.

[10] Reinhart K., Kuhn H.J., Hartog C., Bredle DonaldL (2004). Continuous central venous and pulmonary artery oxygen saturation monitoring in the critically ill. Intensive Care Med.

[11] Futier E., Robin E., Jabaudon M. (2010). Central venous O2 saturation and venous-to-arterial CO2 difference as complementary tools for goal-directed therapy during high-risk surgery. Crit Care.

[12] Crowley R., Sanchez E., Ho J.K. (2011). Prolonged Central Venous Desaturation Measured by Continuous Oximetry Is Associated with Adverse Outcomes in Pediatric Cardiac Surgery. Anesthesiology.

[13] Ranucci M., Isgrò G., De La, Torre T. (2008). Continuous Monitoring of Central Venous Oxygen Saturation (Pediasat) in Pediatric Patients Undergoing Cardiac Surgery: A Validation Study of a New Technology. J Cardiothorac Vasc Anesth.

[14] Ferreira FMC. Terapia hemodinâmica guiada por saturação venosa central contínua em pacientes pediátricos submetidos à cirurgia cardíaca ensaio clínico randomizado. 2018.

[15] Tapia P., Soto D., Bruhn A. (2015). Impairment of exogenous lactate clearance in experimental hyperdynamic septic shock is not related to total liver hypoperfusion. Crit Care.

[16] Bakker J., Postelnicu R., Mukherjee V. (2020). Lactate. Crit Care Clin.

[17] García Mancebo J., De La Mata Navazo S., López-Herce Arteta E. (2021). A comparative two-cohort study of pediatric patients with long term stay in ICUs. Sci Rep.

[18] Kocsi S., Demeter G., Fogas J., Érces D., Kaszaki J., Molnár Z. (2012). Central venous oxygen saturation is a good indicator of altered oxygen balance in isovolemic anemia. Acta Anaesthesiol Scand.

[19] Walley KR. (2011). Use of Central Venous Oxygen Saturation to Guide Therapy. Am J Respir Crit Care Med.

[20] Pastene B., Bernat M., Baumstark K. (2023). OCOSO2: study protocol for a single-blinded, multicentre, randomised controlled trial assessing a central venous oxygen saturation-based goal-directed therapy to reduce postoperative complications in high-risk patients after elective major surgery. Trials.

[21] Molnar Z., Nemeth M. (2018). Monitoring of Tissue Oxygenation: an Everyday Clinical Challenge. Front Med.

[22] Epstein R., Ohliger S.J., Cheifetz I.M., Malay S., Shein SL. (2022). Trends in Time to Extubation for Pediatric Postoperative Cardiac Patients and Its Correlation with Changes in Clinical Outcomes: A Virtual PICU Database Study*. Pediatr Crit Care Med.

[23] Harris K.C., Holowachuk S., Pitfield S. (2014). Should early extubation be the goal for children after congenital cardiac surgery?. J Thorac Cardiovasc Surg.

[24] Meng B., Wu K., Wang Y., Zhang S., Zhou X., Ding Y. (2020). Effect of retrograde autologous priming based on miniaturized cardiopulmonary bypass in children undergoing open heart surgery: A STROBE compliant retrospective observational study. Medicine (Baltimore).

[25] Redlin M., Koster A., Huebler M. (2008). Regional differences in tissue oxygenation during cardiopulmonary bypass for correction of congenital heart disease in neonates and small infants: Relevance of near-infrared spectroscopy. J Thorac Cardiovasc Surg.

[26] Dykes P.C., Lowenthal G., Lipsitz S. (2022). Reducing ICU Utilization, Length of Stay, and Cost by Optimizing the Clinical Use of Continuous Monitoring System Technology in the Hospital. Am J Med.

[27] Reinhart K., Bloos F. (2005). The value of venous oximetry. Curr Opin Crit Care.

[28] Nicklas J.Y., Diener O., Leistenschneider M. (2020). Personalised haemodynamic management targeting baseline cardiac index in high-risk patients undergoing major abdominal surgery: a randomised single-centre clinical trial. Br J Anaesth.

[29] Cheng X qi, Zhang J yan, Wu H. (2020). Outcomes of individualized goal-directed therapy based on cerebral oxygen balance in high-risk patients undergoing cardiac surgery: A randomized controlled trial. J Clin Anesth.

[30] Saugel B., Vincent J.L., Wagner JY. (2017). Personalized hemodynamic management. Curr Opin Crit Care.

